# Effect of depressive symptoms on quality of work life in female nurses: a cross-sectional study using propensity score matching

**DOI:** 10.3389/fpsyt.2023.1213025

**Published:** 2023-09-13

**Authors:** Jia-Ning Li, Xiao-Qian Chen, Qing-Qing Li, Qing-Xiang Zheng, Yu-Qing Pan, Ling Huang, Yu Zhu, Ru-Lin Liu, Xiu-Min Jiang

**Affiliations:** ^1^School of Nursing, Fujian Medical University, Fuzhou, Fujian, China; ^2^Fujian Maternity and Child Health Hospital College of Clinical Medicine for Obstetrics and Gynecology and Pediatrics, Fujian Medical University, Fuzhou, Fujian, China; ^3^Fujian Obstetrics and Gynecology Hospital, Fuzhou, Fujian, China; ^4^School of Public Health, Fujian Medical University, Fuzhou, Fujian, China; ^5^School of Nursing, Fujian University of Traditional Chinese Medicine, Fuzhou, Fujian, China

**Keywords:** depressive symptoms, female nurses, propensity score matching, quality of work life, cross-sectional study

## Abstract

**Background:**

Female nurses have been considered as a vulnerable population in the context of mental health, due to the nature of their work, which can be stressful and emotionally taxing. Understanding the relationship between depressive symptoms and quality of work life (QWL) can contribute to improving mental health and job performance. However, limited studies have focused on the effect of depressive symptoms on QWL in female nurses.

**Objectives:**

The present study aimed to assess the effect of depressive symptoms on female nurses’ QWL using propensity score matching (PSM).

**Methods:**

A cross-sectional, online study using convenience sampling was conducted among 1,401 female nurses in China. PSM was used to minimize the impact of potential confounders between no depressive symptoms and depressive symptoms. Stepwise multiple linear regression analyses were performed on the PSM samples to explore the effects of depressive symptoms on the QWL.

**Results:**

The results revealed there were 33.5% of the female nurses reported depressive symptoms before PSM. And female nurses in this study had a moderate level of QWL before PSM (122.11 ± 18.15), which remained steady after PSM (118.33 ± 18.04). After PSM, the final sample contained 864 female nurses. Stepwise multiple linear regression results indicated that depressive symptoms were the most strongly associated with QWL (*β* = −0.454, *p* < 0.001).

**Conclusion:**

This study highlights the importance of developing mental health plans and psychological interventions for female nurses to maintain mental health and QWL, which is critical to the nursing workforce’s sustainability.

## 1. Introduction

According to the World Health Organization’s projections, depression is anticipated to become the leading contributor to the global disease burden by 2030 (1). Approximately 350 million people suffer from depression worldwide (2). Compared with the general population, nurses are particularly susceptible to developing depression due to exposure to emotional and traumatic situations (3). Female nurses are particularly vulnerable, with a higher likelihood of suffering from depression than males (4). Depression among nurses with a prevalence ranging from 22 to 35% (5–7). Depressive symptoms can limit nurses’ psychosocial functioning, reduce their quality of life, and be unfavorable to their care work (8). Additionally, depressive symptoms in females may exhibit more intricate characteristics as a result of several contributing factors including physiological hormones and external stimuli (9). Therefore, female nurses’ depressive symptoms should also be recognized and addressed as a significant public health concern, as it is vital to ensure the formation of an emotionally healthy nursing workforce.

Quality of work life (QWL) is a worker’s satisfaction with the working life (10), which is characterized by the relationship between the worker and the working environment (11). Specifically, in the field of nursing, it pertains to the degree to which nurses can fulfill their personal needs (e.g., physical, emotional, and social) through their work environment experience and achieve organizational goals (12). Female nurses are consistently subjected to elevated expectations and take on a variety of dual roles at home and work (13). High QWL is relevant to alleviating the family-work conflict (14). Besides, enhancing the QWL for nurses is crucial for stabilizing the nursing workforce, especially in countries like China where the nurse-to-population ratio (per thousand population is 3.56) is relatively low compared to Western countries (15). Furthermore, given that female nurses constitute 97% of the over five million nursing workforce (16), healthcare managers have placed significant emphasis on improving their QWL, as poor QWL may lead to staff turnover, exacerbating the shortage of nursing human resources and cannot meet the growing demand for medical services (17, 18).

However, the effect of depressive symptoms on QWL among female nurses remains unclear. Unexplored associations between depressive symptoms and QWL may limit a variety of interventions targeting nurses’ mental health and planning aimed at the long-term development of national nursing careers. Additionally, propensity score matching (PSM) is a valuable statistical method that minimizes selection bias and confounding variables in observational studies. This methodology strives to emulate the characteristics of a randomized controlled study, allowing for a more reasonable comparison between exposure group and the control group (19). Building upon our primary focus on the main effect, we have meticulously considered a comprehensive range of factors. These include participants’ individual characteristics (such as age, BMI, educational level, etc.), work-related factors (such as employment type, weekly working hours, shift work schedules, etc.), as well as lifestyle and health-related factors (such as sleep quality, physical activity, menstruation status, etc.) which are well-known potential factors of depressive symptoms and QWL (20, 21). Therefore, this study aimed to explore the effect of depressive symptoms on QWL in female nurses through PSM adjusting for confounding variables, which would provide inspiration for managers to developing mental health plans and psychological interventions for female nurses to improve mental health and QWL, then maintaining the nursing workforce.

### 1.1. Theoretical framework

Job Demands–Resources theory (J-DR) guided this study (22). This model proposes that high job demand (e.g., emotional labor, physical demands, shift work, etc.) and low job resources (e.g., social support, team collaboration, training and development, etc.) can dampen worker motivation and exacerbate strain (23, 24), which can have negative effects on QWL. In this study, the main mechanism by which depressive symptoms may have an impact on the QWL is by depleting people’s resources while adding additional demands. On one hand, depressive symptoms may impair concentration and judgment at work, further diminishing the individual’s ability to cope with the demands of the job. On the other hand, individuals affected by depressive symptoms may lack behaviors such as actively seeking social support and increasing self-efficacy, which reduces their ability to access and utilize work resources. At the same time, depressive symptoms are closely linked to health risk behaviors, such as insufficient physical activity and sleep reduction (25, 26), which reduces an individual’s access to psychological and physical resources. These behaviors play an important role in the onset of depressive symptoms and need to be evaluated as explaining or confounding factors. Based on this, the following hypothesis was posited: depressive symptoms have a negative impact on the QWL for female nurses.

## 2. Materials and methods

### 2.1. Study design, setting, and participants

This cross-sectional, descriptive study was conducted on 1,485 female nurses working in 4 hospitals in Fujian province, China from December 2022 to January 2023. To ensure uniformity, four investigators from each participating hospital underwent comprehensive training on the study protocol. Data collection was conducted online through WenJuanXing, a widely employed professional questionnaire survey platform in China. Participants who indicated their willingness to take part in the study were directed to an online informed consent form and the questionnaire through a Quick Response (QR) code provided by the four investigators. Subsequently, participants completed the informed consent process and responded to the questionnaire electronically. The survey was conducted following the Strengthening the Reporting of Observational Studies in Epidemiology (STROBE) statement (27).

The study employed inclusion and exclusion criteria. Eligible participants were required to meet the following inclusion criteria: (a) possession of professional qualification certificates; (b) female gender; and (c) voluntary agreement to participate in this study. The exclusion criteria were as follows: (a) retired nurses, refresher nurses, or student nurses; (b) work less than 6 months; and (c) absence due to illness, marriage, maternity, or other personal reasons for more than 1 month. The sample size was determined based on a previous study (28), where the standard deviation (S) for QWL equaled to σ was 0.6 and permissible error δ was 0.06. Using the formula *N*= $\frac{\left(Z_{1-\alpha /2}\times \sigma \right)}{\delta^{2}}$ for a cross-sectional survey to calculate sample size (α = 0.05; $Z_{1-\alpha /2}$ =1.96), it was determined that a minimum sample size of 385 was required.

### 2.2. Measures

#### 2.2.1. Demographic characteristics

Data on participants’ demographic information was collected, including age, body mass index (BMI), educational level, professional rank, employment type, personal monthly income, marital status, menstruation status, weekly working hours, and shift work schedule.

#### 2.2.2. The short version of the international physical activity questionnaire

The IPAQ-SF is a 7-item instrument assessing physical activity. The Chinese version of IPAQ-SF has been validated among Chinese university students (29), measuring the intensity, frequency, and duration of physical activity over the last 7 days. Participants’ physical activities were classified into three categories of physical activity (low, moderate, and high), which were calculated on the metabolic equivalent of tasks (METs). METs consider the type of activity, such as walking, moderate physical activity, and vigorous physical activity, along with the time spent on each activity. Following the IPAQ guidelines, researchers can determine the level of physical activity by calculating the MET score.

#### 2.2.3. The Pittsburgh sleep quality index

The PSQI is a self-reported instrument that comprises 19 items, which measure an individual’s sleep quality for the preceding month (30). The PSQI assesses seven components, including subjective sleep quality, sleep latency, sleep duration, habitual sleep efficiency, sleep disturbances, hypnotic use, and daytime dysfunction. Each item is rated on a 4-point Likert scale (0 = normal, 1 = mild dysfunction, 2 = moderate dysfunction, and 3 = severe dysfunction). The total scores of seven components are summed up to generate a global score, which ranges from 0 to 21, with a higher score indicating poorer sleep quality. The Chinese version of the PSQI has well-established overall reliability (*r* = 0.82–0.83) and test–retest reliability (*r* = 0.85) (31). Additionally, the critical value of seven points could better differentiate between good and poor sleepers among the Chinese population (32). In this study, Cronbach’s α coefficient of this scale was 0.76.

#### 2.2.4. Self-rating depression scale

The SDS is a 20-item instrument assessing the depressive symptoms of participants (33). The SDS is considered a reliable tool for assessing depressive symptoms in the Chinese population (34), with high internal consistency (Cronbach’s α = 0.89). Responses could range from 1 (never) to 4 (always), with 10 items on positive symptoms and 10 items on negative symptoms. The total score ranged from 25 to 100 and was obtained by multiplying the total original score by 1.25, with higher scores indicating more severe depressive symptoms. In China, a cut-off score of 53 is considered an indicator of risk for clinical depression, based on which the level of depressive symptoms was classified as mild (53–62 points), moderate (63–72 points), and severe (>72 points). In this study, Cronbach’s α coefficient of this scale was 0.88.

#### 2.2.5. The work-related quality of life-2 scale

The WRQOL-2 scale is a 34-item instrument evaluating respondents’ QWL, which was developed by Van Laar et al. (35), and translated and revised by Shao et al. (36). The instrument measures 7 dimensions of the working conditions (WCS), stress at work (SAW), control at work (CAW), homework interface (HWI), employment evaluation of nurse (EEN), general well-being (GWB), and job and career satisfaction (JCS), using a 5-point scale scoring method to rate agreement levels for each dimension (1 = “Strongly disagree” to 5 = “Strongly agree”). Before analysis, negatively worded items for SAW were reverse-scored. The Chinese version of WRQOL-2 scale consists of 32 scoring items, with a total score ranging from 32 to 160. Higher scores indicate higher QWL. The Chinese version of the instrument exhibited robust reliability and validity when utilized within the nursing profession in mainland China, as evidenced by a Cronbach’s α coefficient of 0.94 (37). In this study, Cronbach’s α coefficient of this scale was 0.96.

### 2.3. Statistical analyses

The statistical analyses were performed using IBM SPSS, version 27.0, and R, version 4.1.3. PSM was utilized to adjust for potential confounding variables between female nurses with no depressive symptoms and depressive symptoms. PSM draws more accurate conclusions about the association between depressive symptoms and QWL. The PSM matched age, BMI, educational level, professional rank, employment type, personal monthly income, marital status, menstruation status, weekly working hours, shift work schedule, sleep quality, and physical activity. PSM was performed at 1:1 nearest-neighbor with a caliper value of 0.05 using logistic regression.

The distributions of continuous variables such as QWL are described as means and standard deviations, and the distributions of categorical variables are expressed as frequencies and percentages. Baseline characteristics were compared across no depressive symptoms and depressive symptoms groups using Chi-squared tests for categorical variables and independent *t*-tests for continuous variables. Stepwise multiple linear regression analysis was conducted on the PSM samples to analyze the effect of depressive symptoms on QWL. All statistical tests were two-sided tests, and a *p* value of <0.05 was considered statistically significant.

### 2.4. Ethical considerations

The study was approved by the Ethical Committee of Fujian Maternity and Child Health Hospital (No. 2022YJ071). Regarding online survey consent, the initial section of the questionnaire mainly included informed consent. After reading the consent information, participants had to click on a response button “I agree to participate,” indicating that they had read the consent information and agreed to participate before being allowed to complete the online questionnaire. The questionnaire survey was anonymous and participants had the right to withdraw from the study at any time and for any reason. All methods in this study have been carried out following relevant guidelines and regulations. All data collected were confidential and used only in this study.

## 3. Results

### 3.1. Participant characteristics

A total of 1,485 questionnaires were distributed among female nurses, out of which 1,401 questionnaires were considered valid after eliminating inaccurate data or quality issues. The effective recovery rate was determined to be 94.3%. Among 1,401 female nurses, approximately two-thirds of participants were contract employees (*n* = 932). The age distribution of the participants showed that those aged 26–35 years accounted for the highest percentage (*n* = 573, 40.9%). In terms of marital status, the majority of participants were married (*n* = 759, 54.2%). A significant proportion of participants had an intermediate level of regular menstruation (*n* = 889, 63.5%) or held a senior nurse title (*n* = 600, 42.8%), while almost all had attained a junior college degree or higher qualification (*n* = 1,325, 94.6%). Additionally, 42.7% of participants reported engaging in high levels of physical activity (*n* = 598), while over half of the participants reported having poor sleep quality (*n* = 816).

### 3.2. Comparison of no depressive symptoms and depressive symptoms groups before and after PSM

**Figure 1:**
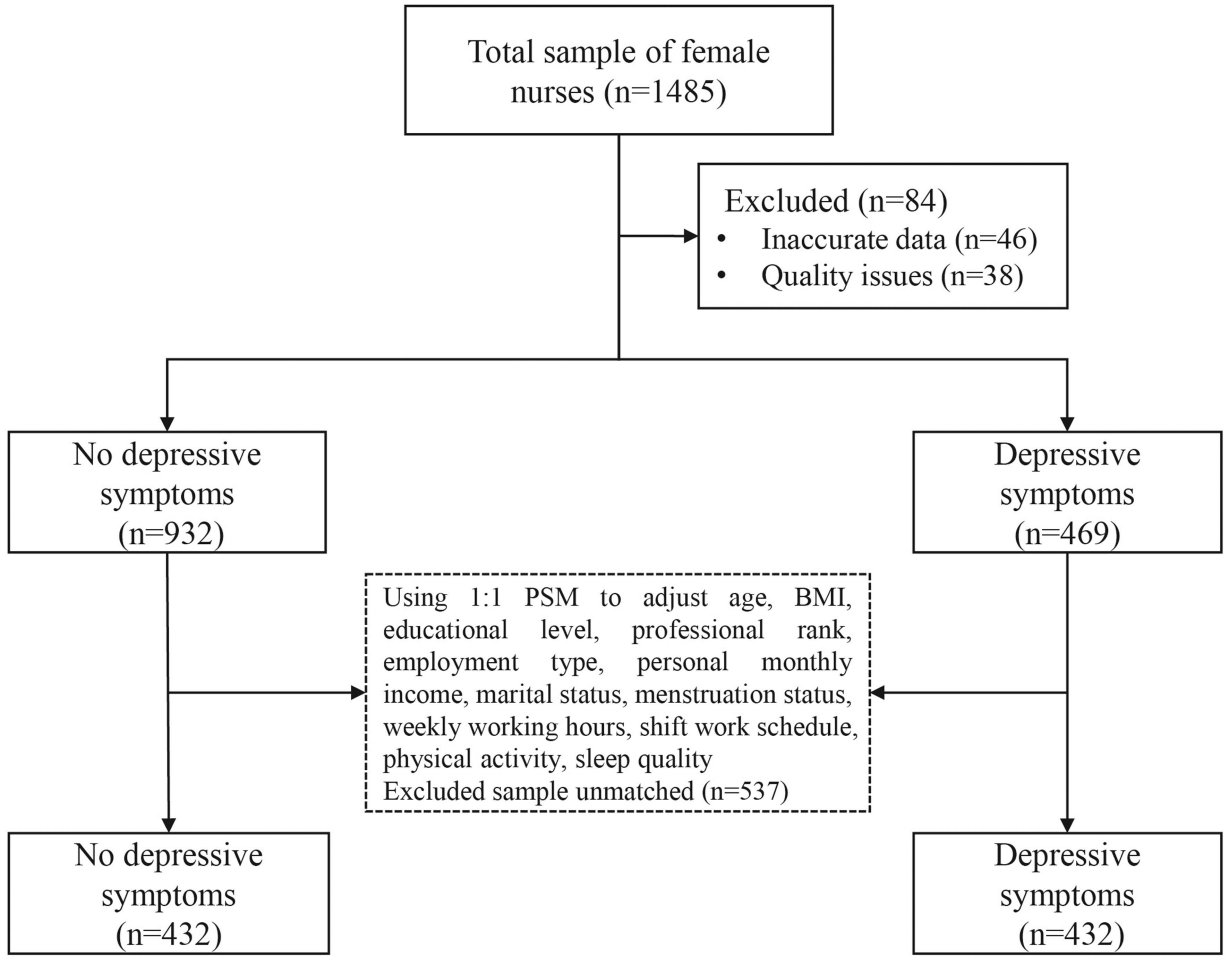
Flowchart of this study. BMI, body mass index; PSM, propensity score matching.

**Figure 2:**
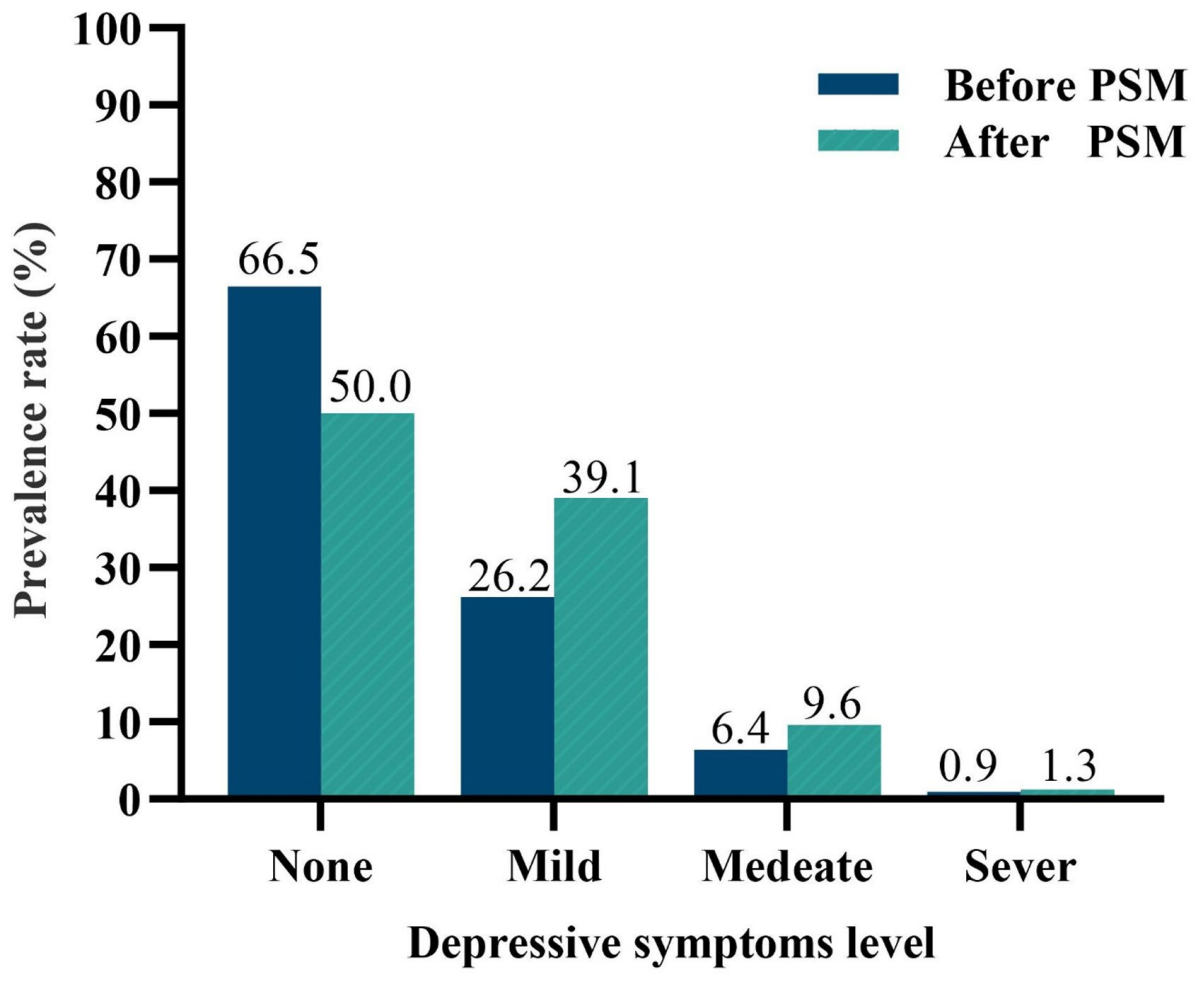
Comparison of depressive symptoms level before and after PSM.

The study flowchart is depicted in Figure 1. After PSM, the sample size was reduced from 1,401 to 864, with an even distribution of 432 participants in each group. Depressive symptoms levels are shown in Figure 2, there were 33.5 and 50% of the female nurses reported depressive symptoms before and after PSM according to the SDS scale, respectively. Table 1 displays the data for no depressive symptoms and depressive symptoms groups before and after PSM. The PSM eliminated confounding bias, making the no depressive symptoms and depressive symptoms groups comparable. After PSM, there were no statistically significant differences between the two groups in age, BMI, educational level, professional rank, employment type, personal monthly income, marital status, menstruation status, weekly working hours, shift work schedule, and sleep quality (*p* > 0.05). Nevertheless, physical activity still shows a significant difference (*p* < 0.05).

**Table 1:** Comparison of no depressive symptoms and depressive symptoms groups before and after PSM.

Variables	Before PSM (*n* = 1,401)		*χ* ^2^	*p*	After PSM (*n* = 864)		*χ* ^2^	*p*
	No depressive symptoms (*n* = 932)	Depressive symptoms (*n* = 469)			No depressive symptoms (*n* = 432)	Depressive symptoms (*n* = 432)		
Age			6.744	0.081			6.610	0.085
≤25	355 (38.09)	166 (35.39)			174 (40.28)	154 (35.65)		
26–35	369 (39.59)	204 (43.5)			164 (37.96)	187 (43.29)		
36–45	166 (17.81)	89 (18.98)			75 (17.36)	82 (18.98)		
≥46	42 (4.51)	10 (2.13)			19 (4.4)	9 (2.08)		
Body mass index			1.870	0.393			1.019	0.601
Underweight	163 (17.49)	92 (19.62)			88 (20.37)	79 (18.29)		
Normal	644 (69.1)	324 (69.08)			301 (69.68)	303 (70.14)		
Overweight/obese	125 (13.41)	53 (11.3)			43 (9.95)	50 (11.57)		
Educational level			5.551	0.136			0.822	0.859^a^
Technical secondary school degree	42 (4.51)	34 (7.25)			21 (4.86)	26 (6.02)		
Junior college degree	490 (52.58)	251 (53.52)			232 (53.7)	229 (53.01)		
Bachelor degree	394 (42.27)	182 (38.81)			176 (40.74)	175 (40.51)		
Master degree and above	6 (0.64)	2 (0.43)			3 (0.69)	2 (0.46)		
Professional rank							5.969	0.113
Junior nurse	382 (40.99)	186 (39.66)	5.909	0.116	186 (43.06)	168 (38.89)		
Senior nurse	386 (41.42)	214 (45.63)			173 (40.05)	201 (46.53)		
Assistant advanced nurse	138 (14.81)	64 (13.65)			61 (14.12)	58 (13.43)		
Associate advanced nurse or advanced nurse	26 (2.79)	5 (1.07)			12 (2.78)	5 (1.16)		
Employment type			1.742	0.187			0.005	0.942
Formal employees	323 (34.66)	146 (31.13)			142 (32.87)	141 (32.64)		
Contract employees	609 (65.34)	323 (68.87)			290 (67.13)	291 (67.36)		
Personal monthly income (yuan)			27.227	**<0.001**			1.357	0.852
<3,000	17 (1.82)	21 (4.48)			11 (2.55)	14 (3.24)		
3,000–5,999	228 (24.46)	151 (32.2)			138 (31.94)	126 (29.17)		
6,000–8,999	350 (37.55)	172 (36.67)			168 (38.89)	167 (38.66)		
9,000–11,999	235 (25.21)	99 (21.11)			90 (20.83)	99 (22.92)		
≥12,000	102 (10.94)	26 (5.54)			25 (5.79)	26 (6.02)		
Marital status			0.215	0.643			0.042	0.838
Unmarried	423 (45.39)	219 (46.7)			199 (46.06)	202 (46.76)		
Married	509 (54.61)	250 (53.3)			233 (53.94)	230 (53.24)		
Menstruation status			35.391	**<0.001**			0.118	0.732
Irregular	290 (31.12)	222 (47.33)			187 (43.29)	192 (44.44)		
Regular	642 (68.88)	247 (52.67)			245 (56.71)	240 (55.56)		
Weekly working hours (hour)			11.989	**0.007**			3.605	0.307
≤35	231 (24.79)	100 (21.32)			90 (20.83)	95 (21.99)		
36–40	528 (56.65)	251 (53.52)			250 (57.87)	235 (54.4)		
41–45	151 (16.2)	94 (20.04)			83 (19.21)	84 (19.44)		
≥46	22 (2.36)	24 (5.12)			9 (2.08)	18 (4.17)		
Shift work schedule			6.651	0.084			3.093	0.378
Forward-rotating night shift	68 (7.3)	40 (8.53)			38 (8.8)	35 (8.1)		
Backward-rotating night shift	453 (48.61)	246 (52.45)			211 (48.84)	224 (51.85)		
12-hour rotating night shift	145 (15.56)	79 (16.84)			67 (15.51)	77 (17.82)		
Day shift	266 (28.54)	104 (22.17)			116 (26.85)	96 (22.22)		
Physical activity			10.034	**0.007**			9.187	**0.010**
Low	99 (10.62)	75 (15.99)			43 (9.95)	63 (14.58)		
Moderate	438 (47)	191 (40.72)			222 (51.39)	181 (41.9)		
High	395 (42.38)	203 (43.28)			167 (38.66)	188 (43.52)		
Sleep quality			106.358	**<0.001**			0.000	1.000
Good sleep	479 (51.39)	106 (22.6)			106 (24.54)	106 (24.54)		
Poor sleep	453 (48.61)	363 (77.4)			326 (75.46)	326 (75.46)		

Bold values were statistically significant. ^a^Fisher exact test.

### 3.3. Comparison of QWL between no depressive symptoms and depressive symptoms groups

The results indicated that the overall female nurses’ QWL was at a moderate level before PSM (122.11 ± 18.15), which remained consistent after PSM (118.33 ± 18.04). Of the seven QWL dimensions, the “stress at work” dimension had the lowest levels before (15.84 ± 3.74) and after (15.14 ± 3.61) the PSM. Table 2 shows a comparison of QWL scores among female nurses before and after PSM in no depressive symptoms and depressive symptoms groups. However, there was a significant difference in the QWL and its dimensions scores between the no depressive symptoms and depressive symptoms groups. The use of PSM helped to ensure comparability between the two groups, thereby strengthening the validity of the results.

**Table 2:** Comparison of quality of work life between no depressive symptoms and depressive symptoms groups before and after PSM.

Variables	Before PSM (*n* = 1,401)		*t*	*p*	After PSM (*n* = 864)		*t*	*p*
	No depressive symptoms (*n* = 932)	Depressive symptoms (*n* = 469)			No depressive symptoms (*n* = 432)	Depressive symptoms (*n* = 432)		
Quality of work life (potential point: 32–160)	128.29 ± 14.89	109.82 ± 17.79	19.33	<0.001	126.51 ± 14.49	110.15 ± 17.53	14.95	<0.001
Working conditions (potential point: 6–30)	25.00 ± 3.23	21.49 ± 4.38	15.42	<0.001	24.67 ± 3.13	21.57 ± 4.33	12.04	<0.001
Stress at work (potential point: 5–25)	16.85 ± 3.47	13.84 ± 3.44	15.37	<0.001	16.46 ± 3.32	13.82 ± 3.4	11.57	<0.001
Control at work (potential point: 5–25)	20.45 ± 2.89	17.82 ± 3.79	13.19	<0.001	20.28 ± 2.77	17.88 ± 3.67	10.85	<0.001
Homework interface (potential point: 2–10)	8.52 ± 1.18	7.30 ± 1.72	13.74	<0.001	8.41 ± 1.17	7.33 ± 1.69	10.96	<0.001
Employment evaluation of nurse (potential point: 5–25)	20.90 ± 2.76	18.00 ± 3.79	14.72	<0.001	20.69 ± 2.69	18.10 ± 3.71	11.77	<0.001
General well-being (potential point: 5–25)	20.17 ± 2.89	16.99 ± 3.73	16.2	<0.001	19.70 ± 2.87	17.01 ± 3.72	11.93	<0.001
Job and career satisfaction (potential point: 4–20)	16.39 ± 2.15	14.38 ± 2.74	13.89	<0.001	16.29 ± 2.05	14.44 ± 2.71	11.34	<0.001

### 3.4. Stepwise multiple linear regression analysis for the factors of QWL

Table 3 presents the results of the stepwise multiple linear regression analyses, which largely confirmed the hypothetical model. Although different independent variables were included in each regression model, depressive symptoms consistently had the strongest association with QWL (*β* = −0.454, *p* < 0.001) and its seven dimensions, including WCS (*β* = −0.379, *p* < 0.001), SAW (*β* = −0.364, *p* < 0.001), CAW (*β* = −0.346, *p* < 0.001), HWI (*β* = −0.345, *p* < 0.001), EEN (*β* = −0.371, *p* < 0.001), GWB (*β* = −0.374, *p* < 0.001), and JCS (*β* = −0.366, *p* < 0.001).

**Table 3:** Stepwise multiple linear regression analysis for the factors of quality of work life and its dimensions after PSM (*n* = 864).

Dependent variable	Independent variables	*B*	SE	*β*	*t*	*p*
Quality of work life^a^	Depressive symptoms	−16.356	1.081	−0.454	−15.132	<0.001
	Poor sleep quality	−5.117	1.258	−0.122	−4.068	<0.001
	Weekly working hours (41–45 h)	−3.256	1.371	−0.071	−2.376	0.018
Working conditions^b^	Depressive symptoms	−3.093	0.256	−0.379	−12.075	<0.001
	Poor sleep quality	−0.728	0.298	−0.077	−2.447	0.015
Stress at work^c^	Depressive symptoms	−2.626	0.222	−0.364	−11.847	<0.001
	Poor sleep quality	−1.515	0.260	−0.181	−5.832	<0.001
	Weekly working hours (41–45 h)	−0.947	0.282	−0.104	−3.361	0.001
	Personal monthly income (9,000–11,999 yuan)	−0.715	0.275	−0.082	−2.603	0.009
	Married	0.458	0.226	0.063	2.022	0.043
Control at work^d^	Depressive symptoms	−2.392	0.220	−0.346	−10.853	<0.001
	Regular menstruation	0.506	0.222	0.073	2.280	0.023
Homework interface^e^	Depressive symptoms	−1.067	0.098	−0.345	−10.850	<0.001
	Weekly working hours (41–45 h)	−0.273	0.125	−0.069	−2.181	0.029
	Weekly working hours (≥46 h)	−0.733	0.284	−0.082	−2.577	0.010
	Poor sleep quality	−0.241	0.115	−0.067	−2.102	0.036
Employment evaluation of nurse^f^	Depressive symptoms	−2.589	0.219	−0.371	−11.823	<0.001
	Poor sleep quality	−0.730	0.256	−0.090	−2.852	0.004
	Regular menstruation	0.477	0.222	0.068	2.150	0.032
General well-being^g^	Depressive symptoms	−2.677	0.222	−0.374	−12.060	<0.001
	Poor sleep quality	−1.160	0.262	−0.140	−4.422	<0.001
	Personal monthly income (3,000–5,999 yuan)	0.563	0.246	0.072	2.287	0.022
	Weekly working hours (41–45 h)	−0.605	0.283	−0.067	−2.137	0.033
Job and career satisfaction^h^	Depressive symptoms	−1.881	0.163	−0.366	−11.564	<0.001
	Age (26–35 years old)	0.337	0.168	0.064	2.004	0.045
	Poor sleep quality	−0.421	0.190	−0.070	−2.215	0.027
	Personal monthly income (9,000–11,999 yuan)	0.424	0.201	0.068	2.112	0.035

a *F* = 223.652, *p* < 0.001, *R*^2^ = 0.227, Adjusted *R*^2^ = 0.224.

b *F* = 75.897, *p* < 0.001, *R*^2^ = 0.150, Adjusted *R*^2^ = 0.148.

c *F* = 40.543, *p* < 0.001, *R*^2^ = 0.191, Adjusted *R*^2^ = 0.186.

d *F* = 61.785, *p* < 0.001, *R*^2^ = 0.126, Adjusted *R*^2^ = 0.123.

e *F* = 34.109, *p* < 0.001, *R*^2^ = 0.137, Adjusted *R*^2^ = 0.133.

f *F* = 51.554, *p* < 0.001, *R*^2^ = 0.152, Adjusted *R*^2^ = 0.149.

g *F* = 45.353, *p* < 0.001, *R*^2^ = 0.174, Adjusted *R*^2^ = 0.171.

h *F* = 36.152, *p* < 0.001, *R*^2^ = 0.144, Adjusted *R*^2^ = 0.140.

SE, standard error.

## 4. Discussion

In this study, we compared the effect of depressive symptoms on QWL among female nurses through a large sample cross-sectional survey using PSM. To our knowledge, few studies focus on female nurses to explore potential gender-specific aspects of the relationship between depressive symptoms and QWL.

Our study found that the QWL of female nurses was at a moderate level, which is similar to previous studies in the UK (38), Iranian (20), and Canada (39), but higher than in Ghana (40). These similarities and differences across countries may be attributed to variations in cultural, social environments, healthcare systems, and nursing practices, which influence working conditions and subsequently affect nurses’ QWL. It should be noted that international knowledge exchange and globalization standards might be contributing to a convergence in the nursing field, resulting in relatively similar QWL levels among nurses worldwide. Our results also indicated that the scores are slightly higher than those in China (41, 42). This may be due to all participants were from Fuzhou, an area with advanced medical technology and a standardized management system for nurses. Nurses in this region are highly competent and exhibit a high level of job satisfaction and fulfillment. However, it is noteworthy that female nurses had the lowest level in the “stress at work” dimension, meaning female nurses were under pressure. The primary reason may be the multidirectional character and intensity of occupational stressors experienced by nurses. Additionally, the intricate balance required to manage both family and work responsibilities might further amplify the perceived stress among female nurses (13). This phenomenon further underscores the importance of job demands and job resources to enhance mental health and effectively combat stress.

As hypothesized, the QWL of female nurses is negatively affected by their depressive symptoms. Previous studies have explored the impact of mental health on various aspects of nurses’ well-being and reached similar conclusions (43, 44). According to the Job Demands–Resources theory, the depletion of critical job resources within nursing work, which can encompass factors like shift work, exposure to traumatic events, and high levels of stress, is posited to contribute to depressive symptoms (3). Additionally, the strong association between depressive symptoms and various adverse outcomes, including lack of energy, cognitive impairment, circadian variability, sluggishness, and sleep disturbance (45–48). These factors impair concentration and judgment, exacerbating the challenges female nurses face in effectively addressing job demands. Thus, female nurses with depressive symptoms face more social and cognitive demands at work but lack sufficient resources to cope with these demands. This ultimately leads to a decline in the QWL. Therefore, it is crucial to prioritize depressive symptoms of female nurses.

Our finding has identified a concerning trend where over one-third of female nurses are likely to be suffering from depressive symptoms, which exceeds previous study (6). Nurse managers must recognize the complexity of depressive symptoms in female nurses for effective strategies and improved mental health and QWL. To address Job Demands, it’s important to ensure that workload distribution and scheduling take into account the potential impact of mental health issues. Prioritizing shift schedules aligned with circadian rhythms can alleviate strain caused by irregular hours. Nurturing a work-life balance culture assists female nurses in effectively managing their dual roles. To enhance Job Resources, provide female nurses with access to training and professional growth opportunities. Establish peer support groups to facilitate experience-sharing and camaraderie (49). Offer training programs for emotional resilience and stress management (50, 51). Cultivate supportive team environments with regular feedback and recognition systems, enhancing job satisfaction and fostering a sense of accomplishment. Implementing these strategies aligns with the principles of the Job Demands–Resources theory and significantly enhances mental health, job satisfaction, and overall work quality for female nurses.

This study suggests several directions for future research based on a few interesting findings. Firstly, multivariate analysis revealed poor sleep quality also considered a crucial indicator of QWL. Sleep quality and depressive symptoms are often intertwined, and their intricate interaction can exacerbate the adverse effects on QWL. Given that over half of the participants reporting poor sleep, addressing sleep problems is a timely topic for discussion. Hence, future researchers should perhaps also begin to examine the potential mediating mechanism of sleep quality in explaining the influence of depressive symptoms on QWL. Secondly, After PSM, physical activity was still showing a statistically significant difference between the no depressive symptoms and depressive symptoms groups. One explanation is that depressive symptoms may reduce people’s motivation to participate in physical activity. Another explanation is that the onset and development of depression may be related to the fact that physical activity promotes the release of neurotransmitters such as dopamine in the brain, which affects mental health. Further research is needed to better understand the relationship between physical activity and depression. Long-term longitudinal studies could help determine the role of physical activity as a predictor of depression risk, as well as the effect of depression on physical activity levels.

### 4.1. Strengths and limitations

The highlight of this study was focusing on female nurses to determine the effect of depressive symptoms on QWL using a large sample and propensity score matching. However, this study has some limitations that should be considered. Firstly, given the cross-sectional study design, it is challenging to infer causation based on the relationships between depressive symptoms and QWL. Future longitudinal studies are necessary to solve this critical issue. Secondly, the sample size was limited to four hospitals within a single province, which may restrict generalizability. Third, the investigation of female nurses diagnosed with major depression was inadequate since they could have left their jobs due to their illness. Finally, all variables were examined by self-report questionnaires, which may result in response bias.

## 5. Conclusion

This study has implications for better understanding the effect of depressive symptoms on QWL in female nurses using propensity score analysis. This study has demonstrated that depressive symptoms were prevalent among female nurses, and depressive symptoms adversely affect their QWL. These findings highlight the importance of developing mental health plans and implementing psychological interventions as a crucial investment in sustaining the mental health and quality performance of female nurses, thereby maintaining the nursing workforce.

## Data availability statement

The original contributions presented in the study are included in the article, further inquiries can be directed to the corresponding author.

## Ethics statement

The studies involving humans were approved by the Ethical Committee of Fujian Maternity and Child Health Hospital (No. 2022YJ071). The studies were conducted in accordance with the local legislation and institutional requirements. The participants provided their written informed consent to participate in this study.

## Author contributions

J-NL and X-QC: writing—review and editing, conceptualization, methodology, and project administration. X-MJ: project administration and editing, conceptualization, and methodology. Q-QL: writing—original draft and methodology. Q-XZ: writing—review and editing and formal analysis. Y-QP and LH: writing—review and validation. YZ and R-LL: resources and investigation. All authors contributed to the article and approved the submitted version.

## Funding

This study was funded by Social Development Guiding Project Fund from Fujian Science and Technology Department (No. 2023Y0055) and Scientific and Technological Initial Project Fund from Fujian Maternity and Health Hospital (No. YCXH 22-06).

## Conflict of interest

The authors declare that the research was conducted in the absence of any commercial or financial relationships that could be construed as a potential conflict of interest.

## Publisher’s note

All claims expressed in this article are solely those of the authors and do not necessarily represent those of their affiliated organizations, or those of the publisher, the editors and the reviewers. Any product that may be evaluated in this article, or claim that may be made by its manufacturer, is not guaranteed or endorsed by the publisher.
